# Minor shift in background substitutional patterns in the *Drosophila saltans *and *willistoni *lineages is insufficient to explain GC content of coding sequences

**DOI:** 10.1186/1741-7007-4-37

**Published:** 2006-10-18

**Authors:** Nadia D Singh, Peter F Arndt, Dmitri A Petrov

**Affiliations:** 1 Department of Biological Sciences, Stanford University, 371 Serra Mall, Stanford, CA 94305, USA; 2 Max Planck for Molecular Genetics, 14195 Berlin, Germany

## Abstract

**Background:**

Several lines of evidence suggest that codon usage in the *Drosophila saltans *and *D. willistoni *lineages has shifted towards a less frequent use of GC-ending codons. Introns in these lineages show a parallel shift toward a lower GC content. These patterns have been alternatively ascribed to either a shift in mutational patterns or changes in the definition of preferred and unpreferred codons in these lineages.

**Results and discussion:**

To gain additional insight into this question, we quantified background substitutional patterns in the *saltans/willistoni *group using inactive copies of a novel, *Q*-like retrotransposable element. We demonstrate that the pattern of background substitutions in the *saltans/willistoni *lineage has shifted to a significant degree, primarily due to changes in mutational biases. These differences predict a lower equilibrium GC content in the genomes of the *saltans/willistoni *species compared with that in the *D. melanogaster *species group. The magnitude of the difference can readily account for changes in intronic GC content, but it appears insufficient to explain changes in codon usage within the *saltans/willistoni *lineage.

**Conclusion:**

We suggest that the observed changes in codon usage in the *saltans/willistoni *clade reflects either lineage-specific changes in the definitions of preferred and unpreferred codons, or a weaker selective pressure on codon bias in this lineage.

## Background

Codon bias refers to the unequal usage of synonymous codons in protein coding sequences. Preferred codons are believed to correspond to more abundant transfer (t)RNAs [1]. In genes with high codon bias, the frequent presence of preferred codons is believed to increase the efficiency and/or fidelity of translation [2-4]. In *Drosophila*, empirical evidence suggests that the degree of codon bias can have substantial effects on active protein level; replacements of preferred codons with unpreferred ones in the *alcohol dehydrogenase *(*Adh*) gene result in significant decreases in levels of protein activity [5].

Within the genome of a particular species, the level of codon bias for any given gene is thought to be governed by the dynamic interplay among natural selection, mutation, and drift. Accordingly, codon bias levels will be affected by the rate and dominance of mutations affecting codon bias, the stochasticity associated with the population processes affecting these mutations (mediated through factors such as effective population size and recombination rate), and the strength of natural selection on codon bias. Variation in codon usage within a single genome is often associated with variation in one or more of these factors. In *Drosophila melanogaster*, for instance, codon bias is positively correlated with gene expression, which is thought to reflect increased selective benefits of translational efficiency for highly expressed genes [6-8]. Likewise, the negative correlation between codon bias and protein length in this species may result from Hill-Robertson effects [9], or may occur because increases in codon bias have stronger effects in short genes than in long genes [7,10-12]. The increase in codon bias associated with increased recombination rate in *D. melanogaster *is likely due to a recombination-associated substitutional bias [13-15], but Hill-Robertson effects may also play a role [16-18].

The genome hypothesis [19] suggests that the definition of "preferred" and "unpreferred" codons is the same for all genes in the genome, as codon preferences likely reflect tRNA abundances. Changes in codon usage patterns between species may be due to the same forces that modulate codon bias within any particular genome, but may also result from shifts in the definitions of preferred and unpreferred codons. Such shifts are not very common in evolution, and are generally observed only among very distantly related organisms. Within *Drosophila*, for instance, *D. melanogaster *and *D. pseudoobscura*, which diverged 25–40 million years ago (MYA) [20], have nearly identical definitions of major and minor codons [21].

**Figure 1:**
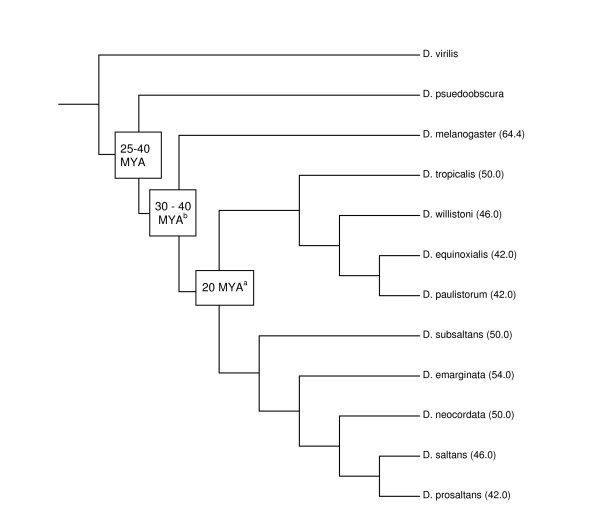
Evolutionary relationships based on *Xdh *among the nine species studied including *D. melanogaster, D. pseudoobscura and D. virilis *as outgroups [26]. GC content at third position sites are presented in parentheses [23, 28]. *D. melanogaster *diverged from *D. pseudoobscura *25–40 MYA [20], *D. melanogaster *and the *saltans/willistoni *groups diverged approximately 30–40 MYA [20], while the *saltans *and *willistoni *groups diverged approximately 20 MYA [27]

In contrast, species in the *D. saltans/willistoni *lineages, which diverged from *D. melanogaster *around 30–40 MYA [20], show a substantially different pattern of codon usage from that found in the *melanogaster *and *obscura *group species. The *saltans/willistoni *lineages diverged from *D. melanogaster *prior to the split of the *melanogaster *and *obscura *groups but after the split of the *Drosophila *and *Sophophora *subgenera (Figure 1). Phylogenetic analysis [22] suggests that species within the *melanogaster *and *obscura *groups show roughly the ancestral pattern of codon usage, while there has been a significant reduction in the usage of G- or C-ending codons in the common ancestor of the *saltans *and *willistoni *lineages. In these species, the reduction of GC content is evident not only at third codon positions but also, albeit to a lesser extent, in introns and at first codon positions [22-26].

One hypothesis for the pronounced change in GC content in coding sequences is that the definitions of preferred and unpreferred codons have changed in the *saltans/willistoni *group [27]. This hypothesis is supported by the observation that some amino acids show a greater change in GC content than do others [27]. However, a change in codon preferences cannot be invoked to explain the change in base composition of noncoding sequences.

One explanation for the concomitant shifts in GC content of codon and noncoding sequences is a genome-wide shift in the pattern of background substitutions [15] in favor of lower GC content in the *saltans/willistoni *group [22,25,28]. Such a shift could arise from either a change in mutational patterns or an increased rate of fixation of As and Ts mediated by biased gene conversion or natural selection. However, it is difficult to reconcile a simple substitutional model with the observed heterogeneity of change in GC content among amino acids.

To help distinguish between these two competing hypotheses, we investigated background patterns of nucleotide substitution in nine species belonging to the *saltans/willistoni *clade. We inferred these patterns by investigating rates of nucleotide substitution in nonfunctional fragments of a novel *Q*-like retrotransposable element. Our results suggest a minor shift in the spectrum of background nucleotide substitution in *saltans/willistoni *group relative to that found in *D. melanogaster*. The magnitude of this shift to is sufficient to generate a reduction of 11.5% at most in the GC content in the *saltans/willistoni *group. This is consistent with the reduction in GC content of intronic sequences in this lineage, but contrasts with the observed reduction in GC content of coding sequences of ~30%. Consequently, we suggest that the change in noncoding GC content in the *saltans/willistoni *group may result from background substitutional patterns, whereas the change in GC content of coding sequences reflects either novel codon preferences or a weakening of selection on codon bias.

## Results

### Identification of *Q-saltans1*

We isolated a novel retrotransposable element in the *D. saltans *genome using degenerate PCR. This element showed highest homology with a *Q *element originally found in the *Anopheles gambiae *genome [29], and accordingly, we named this novel retrotranspon *Q-saltans1*. Using PCR primers specific to *Q-saltans1*, we identified a total of 58 paralogous copies of *Q-saltans1 *from individual flies from nine species in the *saltans *and *willistoni *species groups. Evolutionary relationships among the nine species studied are presented in Figure 1[28]. Ten copies of *Q-saltans1 *were isolated from *D. saltans*, six from *D. prosaltans*, seven from *D. subsaltans*, seven from *D. neocordata*, eight from *D. emarginata*, five from *D. tropicalis*, five from *D. equinoxialis*, five from *D. paulistorum *and five from *D. willistoni*.

### Phylogenetic reconstruction in *D. saltans*

**Figure 2:**
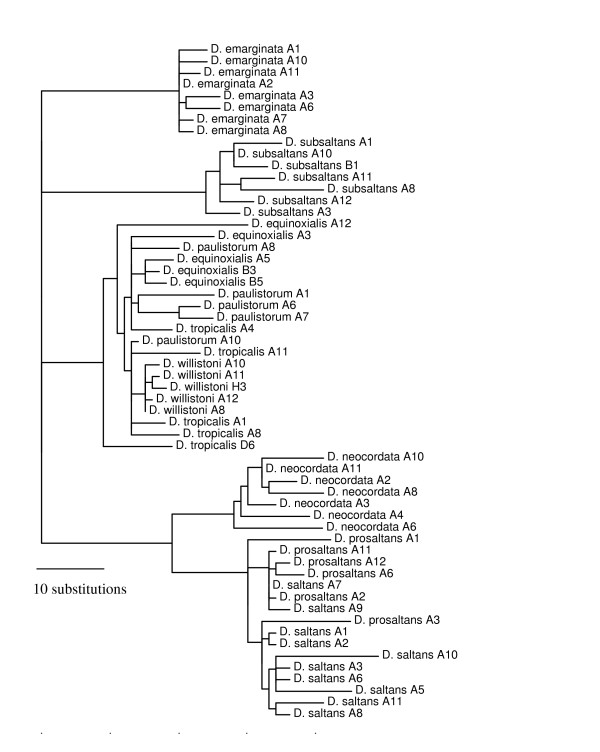
Phylogeny of the fifty-eight paralogous copies of the *Q*-like retrotransposon isolated from the *D. saltans *and *D. willistoni *lineages. Branch lengths are proportional to the number of substitutions incurred along each branch.

We built a phylogenetic tree using parsimony criteria to reconstruct the evolutionary relationships among the 58 paralogs of *Q-saltans1 *(Figure 2). We mapped all 515 unambiguous changes to individual branches of the phylogeny. Changes mapping to the internal branches of the phylogeny are shared among elements, which suggests that they correspond to the constrained evolution of the active lineages of *Q-saltans1 *[30]. Substitutions on terminal branches are unique to individual paralogs and, provided sampling is sufficiently dense, should reflect the neutral evolution of the dead-on-arrival fragments commonly generated by non-long terminal repeat (LTR) elements [30,31]. In this model, substitutions on the internal branches should show substantially more third-position changes compared with the first and second positions, while substitutions on the terminal branches should fall into these classes with equal probability. For internal branches, we did indeed observe a sharp overabundance of third-position changes (81 third-position changes compared with 45 other (first + second) position changes, *P *<< 0.001, G-test). In contrast, we observed 199 other (first + second) changes and 107 third position changes on the terminal branches, which is not significantly different from the expected 2:1 ratio (*P *= 0.67, G-test). We used the identified terminal branch substitutions for all subsequent analyses.

### Intraspecific variability of the rates of nucleotide substitution in *D. melanogaster*

To test for a shift in the pattern of background nucleotide substitution in the *saltans/willistoni *group relative to that of *D. melanogaster*, we compared background substitutional patterns derived from phylogenetic analyses of inactive copies of transposable elements (*Q-saltans1 *in the *saltans/willistoni *group versus the retrotransposable element *Helena *[32] and the non-autonomous DNA element *DNAREP1_DM *[15] in *D*. *melanogaster*). To ensure that comparisons between the patterns estimated from *Q-saltans1 *and the *D. melanogaster *transposable elements truly reflect interspecific differences, we first ascertained the amount of variability in the substitutional patterns that could result from differences in (i) the identity of the elements and their specific sequences, (ii) the genomic location of these transposable elements, and (iii) potential errors of phylogenetic reconstruction.

**Figure 3:**
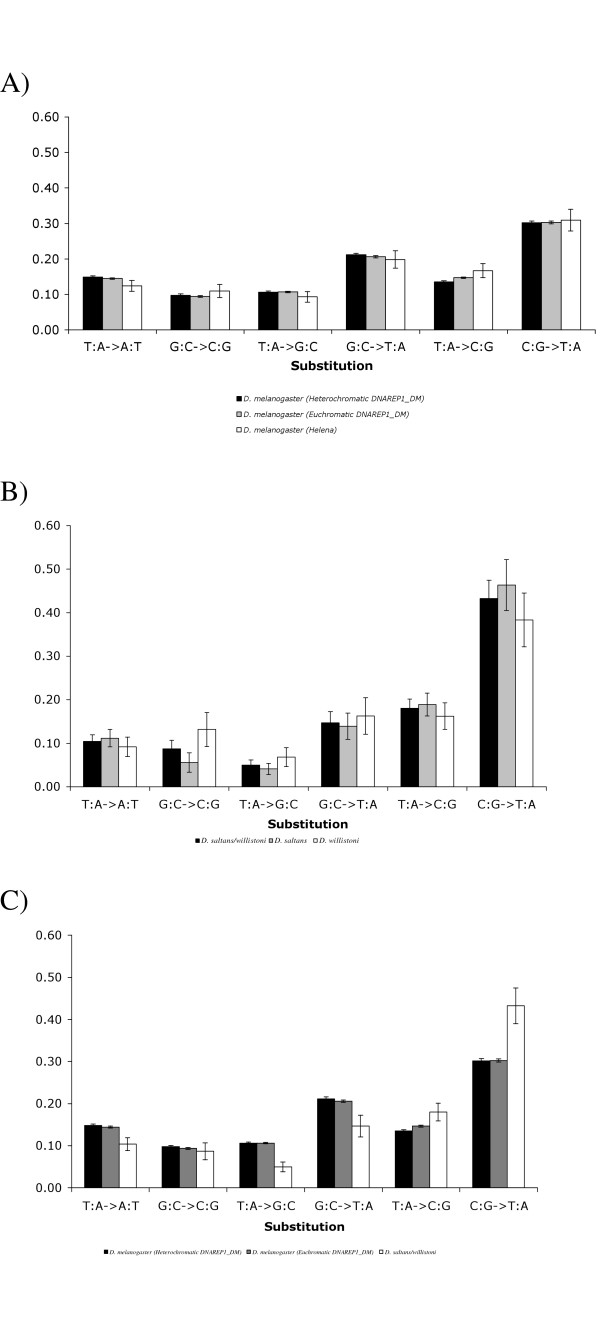
Relative rates of each of the six pairs of complementary nucleotide substitution in A) different noncoding elements from *D. melanogaster*, B) the *saltans/willistoni *group combined and *D. saltans *and *D. willistoni *and separately and C) in *D. melanogaster *and in the combined *saltans/willistoni *group. Three datasets were used to estimate relative rates of nucleotide substitution in *D. melanogaster *(see Materials and Methods). The rate of each pair of complementary nucleotide substitutions is depicted, normalized by the total substitution rate. Error bars represent the standard deviation due to sampling error only.

To assess variation in background substitutional patterns associated with different types of nonfunctional elements and their genomic location, we compared the substitutional profiles inferred from three types of transposable elements in *D. melanogaster *(Figure 3A). Two of the profiles were inferred from patterns of nucleotide substitution in *DNAREP1_DM *[15]; we present the spectrum of single-nucleotide substitutions assuming a star phylogeny for both heterochromatic and euchromatic elements [15]. The third estimate of background substitutional patterns in *D. melanogaster *was inferred from the terminal branch substitutions of the maximum parsimony tree for *Helena *[32]. In each of these three cases, we normalized the estimate of each of the six rates by the estimate of the total substitution rate (which is a function of the mutation rate and of the age of the individual elements), resulting in the estimate of the relative rates of single-nucleotide substitution.

The only significant difference among these three types of data was in the rate of the T:A → C:G transition, which was significantly lower in heterochromatic sequences of *DNAREP1_DM *compared with euchromatic fragments of this element (*P *= 0.002, two-tailed *t*-test). However, the magnitude of the difference in the rate of this transition is quite small (Figure 3A, Table 2). Equilibrium GC content inferred from the patterns of substitutions does vary between heterochromatic and euchromatic sequences of *D. melanogaster*, although the magnitude of the difference is quite small, on the order of 3% [15]. Overall, however, the substitutional profiles estimated using heterochromatic fragments of *DNAREP1_DM*, euchromatic fragments of *DNAREP1_DM*, and nonfunctional fragments of the retrotransposon *Helena *are very similar.

**Figure 4:**
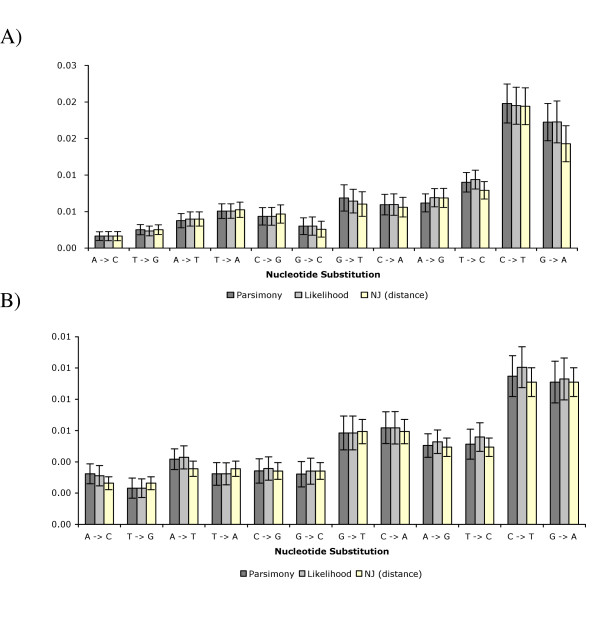
Relative rates of each of the twelve nucleotide substitutions inferred from alternative methods of phylogenetic reconstruction for A) *Q*-like elements in *D. saltans *and *willistoni *and B) *Helena *elements from *D. melanogaster*.

To estimate the sensitivity of the patterns of substitution to errors in phylogenetic reconstruction, we compared rates of nucleotide substitution based on terminal branch substitutions of maximum parsimony, maximum likelihood (HKY85) and neighbor-joining (distance) trees. We explored this potential sensitivity to phylogenetic reconstruction in both the *Q-saltans1 *elements and the *Helena *elements. For both transposable elements, there were no significant differences in rates of individual nucleotide substitutions (*P *> 0.2, all pairwise comparisons, two-tailed *t*-test) (Figure 4A and 4B). In addition, comparisons of equilibrium GC revealed no significant differences (*P *> 0.4, all pairwise comparisons, two-tailed *t*-test).

These results give us confidence that variability in the identity and genomic location of the studied transposable elements or potential errors of phylogenetic reconstructions do not significantly affect estimated patterns of single-nucleotide substitution for these data. Therefore, the comparison of patterns of substitution derived from *Q-saltans1 *and from the *D. melanogaster *transposable elements should primarily reflect interspecific differences in the patterns of nucleotide substitution. In all subsequent analyses, we used the maximum parsimony tree for *Q-saltans1 *and *Helena *and a star phylogeny assumed for *DNAREP1_DM *elements.

### Comparisons of substitutional profiles between *saltans *and *willistoni *lineages

The shift in the pattern of nucleotide substitutions is hypothesized to have occurred prior to the split of the *saltans *and *willistoni *groups. We thus tested whether the patterns of substitution are indeed similar in the *saltans *and *willistoni *species groups. To do so, we identified terminal branch substitutions on the maximum parsimony tree of *Q-saltans1*, and separated those substitutions occurring on branches leading to paralogs isolated from a species in the *saltans *group from those occurring on branches leading to a paralog isolated from a species in the *willistoni *group. These two classes of terminal branch substitutions were then used to infer patterns of substitution specific to the *saltans *and *willistoni *groups, respectively.

The estimated relative rates of single-nucleotide substitutions are presented in Figure 3B. None of the six substitutions was significantly different in pairwise comparisons (*P *> 0.09, all comparisons, two-tailed *t*-test). This homogeneity in background substitutional patterns justifies aggregation of all of the terminal branch substitutions on the *Q-saltans1 *tree to estimate the overall pattern of single-nucleotide substitutions in the *saltans/willistoni *lineage.

### Comparisons of substitutional profiles between *D. melanogaster *and *saltans/willistoni *lineages

For simplicity, we present the comparison of the *saltans/willistoni *substitutional profile with the most robust *D. melanogaster *estimates, those derived from heterochromatic and euchromatic fragments of *DNAREP1_DM*. The resulting profiles are presented in Figure 3C and the comparisons of rates are presented in Table 1. Two nucleotide substitution rates are significantly different between the *D. melanogaster *(both in euchromatin and heterochromatin) and *saltans/willistoni *lineages. The relative rate of T:A → G:C transversion is significantly lower, whereas the rate of C:G → T:A transition is significantly higher in the *saltans*/*willistoni *groups (Bonferroni-corrected *P *<< 0.0001 and *P *< 0.03, respectively, both comparisons, two-tailed *t*-test). In addition, we have tentative support for lower rates of T:A → A:T and G:C → T:A substitutions in the *saltans/willistoni *lineages, although these differences are not significant after correcting for multiple tests (*P *< 0.025, Bonferroni-corrected *P *> 0.05, both comparisons, two-tailed *t*-test). We also estimated predicted equilibrium GC content (GC*) in both lineages based on the rates of nucleotide substitutions, which revealed that GC* in the *saltans *and *willistoni *groups (28.4%) is lower than that in heterochromatic and euchromatic fragments of *D. melanogaster *(32.0% and 33.3%, respectively), although not significantly so (Bonferroni-corrected *P *> 0.1, both comparisons, one-tailed *t*-test). GC* estimated from transitions only, while lower in *D. saltans/willistoni *(29.4%), was not significantly different from comparable estimates in *D. melanogaster *(30.9% and 32.7% in heterochromatic and euchromatic sequences, respectively) (Bonferroni-corrected *P *> 0.2, both comparisons, one-tailed *t*-test).

This analysis was carried out using the combined data from *DNAREP1_DM *fragments taken from low-recombination and high-recombination portions of the euchromatic fraction of the *D. melanogaster *genome. The aggregation of these data is justified given the lack of statistically significant differences in equilibrium base composition between these regions [15]. However, the best estimate of equilibrium GC content in high-recombination regions of *D. melanogaster *euchromatin is higher than that in low-recombination regions, and thus we might be underestimating the magnitude of the shift in GC content in the *saltans/willistoni *lineages. Importantly, equilibrium GC content (GC*) estimated from fragments of *DNAREP1_DM *in regions of high recombination (35.0%) is also not significantly different from GC* in the *saltans/willistoni *lineages (Bonferroni-corrected *P *> 0.05, 1-tailed *t*-test).

### Are differences in mutation rates or differences in the probabilities of fixation responsible for the differences in substitution rates between these lineages?

The observed changes in nucleotide substitutions may result from changes either in the mutation rates or in the probability of fixation of different mutations. To distinguish between these two possibilities, we employed the forward-reverse test [33]. In the case of the comparisons of the *saltans/willistoni *rates with the rates derived from euchromatic *DNAREP1_DM *elements in *D. melanogaster*, the 95% confidence limits of the ratios of the forward and reverse substitutions do allow for the theoretical possibility of the differences in fixation biases explaining the patterns (Table 2). However, the forward-reverse analysis indicates that such differences in fixation probabilities require G:C nucleotides to be very strongly preferred when we consider changes in the rates of transitions (|Nes| > 4 in both lineages) and strongly unpreferred when we consider changes in the rates of transversions (|Nes| > 2 in both lineages) (Table 2). On balance, a shift in mutational patterns appears to be a more likely explanation.

## Discussion

### Estimating patterns of substitution using dead-on-arrival copies of non-LTR elements

Previous data on the base composition of coding and coding sequences suggest that GC content in the *saltans/willistoni *group differs from GC content in *D. melanogaster *[22-25,27,28]. The shift in GC content in coding sequences has been argued to result from novel codon preferences in this species group [27]. However, the observation that base composition of noncoding sequences shows a shift in GC content in the same direction (although to a lesser degree) has been used as evidence in favor of a mutational shift [22,25,28]. To distinguish among these possibilities, we assayed background patterns of nucleotide substitution in unconstrained sequences. This method of using inactive fragments of transposable elements has been used previously to investigate patterns of background point substitution and insertion/deletions in *Drosophila *and other organisms [15,30,34].

To estimate background patterns of single-nucleotide substitutions, we identified a novel non-LTR element in *D. saltans*. We cloned and sequenced 58 paralogous copies of this *Q*-like retrotransposon (*Q-saltans1*) in nine species from the *willistoni *and *saltans *groups. We analyzed substitutions that map to the terminal branches of the phylogenetic trees reconstructed using these sequences, and extracted estimates of the individual rates of single-nucleotide substitutions using a maximum-likelihood procedure [35,36].

The pattern of single-nucleotide substitutions estimated from this procedure conforms to our expectations of unconstrained sequence evolution. Changes were equally likely in all three codon positions, giving us confidence that we indeed observe background patterns of substitution using these sequences. Moreover, our estimates of the spectrum of single-nucleotide substitution are robust to errors in phylogenetic reconstruction. Comparing the profile of background substitutional patterns derived from various methods of phylogenetic reconstruction (maximum parsimony, maximum likelihood, and neighbor-joining) yielded no significant differences; in all cases the estimates of individual rates of single-nucleotide substitutions were virtually identical (*P *> 0.2, all comparisons, two-tailed *t*-test).

### Comparison of the patterns of substitution in dead-on-arrival copies of *Q-saltans1 *and inactive copies of *D. melanogaster *transposable elements

Patterns of background substitution revealed by the analysis of the inactive copies of *Q-saltans1 *appeared similar for *saltans *and *willistoni *groups (Figure 3B), which is consistent with the shift in GC content occurring prior to the separation of these lineages [22,25]. Therefore, we combined all of the *Q-saltans1 *data to estimate the overall pattern of background substitution in the *saltans/willistoni *lineage.

The pattern of nucleotide substitution in *D. melanogaster *was estimated using two different transposable elements: *Helena *[30] and *DNAREP1_DM *[15]. The profiles of single-nucleotide substitutions estimated from these different elements are strikingly similar (Figure 3A), suggesting that the species-specific pattern of background substitution is largely insensitive to the identity of the transposable element studied. While small in magnitude, there do appear to be heterogeneities in background substitutional patterns associated with genomic location [15], and accordingly, we treated euchromatic and heterochromatic copies of *DNAREP1_DM *separately in our analysis.

Overall, the *saltans/willistoni *pattern is quite similar to the patterns estimated in *D. melanogaster *(Figure 3C) and *D. virilis *[30]. In all cases, the most frequent substitution is the C:G → T:A transition, whereas the reverse transition (T:A → C:G) occurs at rates similar to that of transversions [32]. Moreover, the G:C → T:A transversion appears to be most frequent transversions in both *D. melanogaster *and the *saltans/willistoni *group.

However, the *saltans/willistoni *pattern does differ in two respects from the pattern observed in *D. melanogaster*. There is a statistically significant increase in the rate of C:G → T:A transitions and a significant decrease in the rate of the T:A → G:C transversion in the *willistoni/saltans *lineage. Notably, both of these changes lead to a reduction in the overall GC content in *saltans/willistoni *lineages. However, the expected equilibrium GC content based on the *saltans/willistoni *pattern (28.4%) shifts only modestly (roughly 4–7%) compared with that estimated using *DNAREP1_DM *(32.0%, 33.3% and 35.0% for heterochromatic, low-recombination euchromatic, and high-recombination euchromatic sequences, respectively). Given the errors on our estimates of GC* for both species, we can say with 95% confidence that the magnitude of the difference in GC* between these two groups is at most 11.5% and could in fact be nonexistent.

### Distinguishing between mutational generation and fixation probability models

A background substitutional bias can be mediated through a bias either in the generation of novel mutations, or in the fixation probabilities of novel mutations. Note, however, that if the shift toward higher rates of G:C → T:A and C:G → T:A substitutions in the *saltans*/*willistoni *lineages was due entirely to the decrease of the probability of fixation of G:C alleles at the expense of A:T alleles (either due to the effects of natural selection or biased gene conversion), we would then also expect to see a concomitant decrease in the rate of the reverse (T:A → G:C and T:A → C:G) substitutions in the *saltans/willistoni *lineage. However, this does not appear to be the case for the rates of C:G → T:A and T:A → C:G transitions (both of which are increased in the *saltans/willistoni *lineage), or for T:A → G:C and G:C → T:A transversions (both of which are both decreased in the *saltans/willistoni *lineage) (Figure 3C).

We employed a more rigorous test to distinguish between mutational variation and fixation biases for the shift in background substitutional patterns in *D. saltans/willistoni*. The forward-reverse test [33] suggests that while fixation biases can in principle explain the difference in substitutional patterns between *D. melanogaster *and *D. saltans/willistoni*, this model would require novel G:C mutations to be strongly preferred when generated by a transition, while being strongly unpreferred when generated by a transversion. This eliminates the possibility of differences in the strength of natural selection acting on GC content being responsible for these patterns (as it cannot distinguish between A:T versus G:C polymorphisms generated by transitions or transversions). This leaves only the possibility of differences in the strength of biased gene conversion. However, the strength of the putative bias in rates of gene conversion seems extreme and appears implausible, requiring for instance that the rates of forward and reverse mutations be different by over a hundred-fold [33]. Thus, the most probable explanation for the observed differences in substitution patterns in *D. saltans/willistoni *is a change in the rates of mutation.

### Role of novel background substitutional patterns in modulating base composition of coding and noncoding sequences

Although we have documented a minor shift in background substitutional patterns in the *saltans *and *willistoni *species groups, the extent to which this shift is reflected in the base composition of coding and noncoding DNA remains to be seen. Note that, given the divergence time between *D. melanogaster *and *D. saltans/D. willistoni *of approximately 30–40 million years [20] and the rates of neutral point substitution in *Drosophila *[37], we would expect that base composition of unconstrained sequences in *D. saltans *and *D. willistoni *genomes should have approached the equilibrium values corresponding to the derived patterns of point substitution in the *saltans/willistoni *clade.

The documented shift in base composition of intronic sequences is similar between short and long introns, and appears to be minor. For *D. melanogaster*, *D. saltans *and *D. willistoni*, the GC content of short introns is slightly lower than that of long introns (Table 3). Base composition shifts in *D. saltans *and *willistoni *to similar degrees in both intron classes towards ~7% lower GC content in *D. saltans *and ~2% lower GC content in *D. willistoni *[27,38]. The magnitude of this shift in the base composition of intronic sequences is entirely consistent with our estimates of the change in patterns of background substitution, which could generate a difference in GC content of up to ~12%, although it is likely to be smaller.

In contrast, GC content in coding sequences, particularly at third-position sites, in the *saltans/willistoni *group show a much greater depression in GC content relative to *D. melanogaster *than do introns [22-26] (Table 1). On average, GC content at third-position sites in *D. saltans *and *D. willistoni *is 23.6% (13–30%) lower than that at the same sites in *D. melanogaster*. The magnitude of this change in GC content is incompatible with our estimates of background substitutional patterns, which suggests that other evolutionary forces must be at play.

However, we do believe that our documented shift in substitutional biases may serve to modulate base composition at coding sites to some degree. Indeed, it is intriguing that the amino acids with the most significant shifts in codon usage are those two-fold amino acids encoded by C/U-ending codons, as the relative rate of C:G → T:A substitutions has increased significantly in this lineage (Figure 3C). However, given that GC* estimated using all substitutions is very similar to GC* estimated using only transitions, it is not likely that this single pair of complementary nucleotide substitutions is wholly responsible for the exaggerated response of the two-fold degenerate amino acids.

The dramatic shift in base composition in coding sequences might very well reflect novel codon preferences in this species group, as suggested by others [27]. In support of this model is the observation that some amino acids show more marked changes in codon usage than other amino acids in this clade [27], which is seemingly at odds with a mutational model given that almost all preferred codons in *D. melanogaster *are G- or C-ending. In addition, assuming that orthologous genes in *D. melanogaster *and *D. saltans/willistoni *are under similar selection pressures for codon bias, the changing codon preference model predicts that genes with the most highly biased patterns of codon usage will experience the greatest deviation in codon usage patterns relative to *D. melanogaster*, which is precisely what is observed [27].

It is important to note that there are two other forces that may be contributing to the evolution of base composition of coding and noncoding sequences in the *saltans *and *willistoni *lineages. First, transcription-associated mutational biases appear to be operating in several systems (for review see Aguilera [39]). Were such a bias operating in *Drosophila*, this could also shape GC content of genic sequences in this system; however, the concordance between the expected GC content based on background substitutional patterns and the observed GC content of intronic sequences suggests that transcription-associated mutational biases are unlikely to play a significant role in the evolution of base composition of genic sequences in the *saltans/willistoni *lineages. Moreover, comparisons of background substitutional patterns in transcribed and untranscribed sequences in *D. melanogaster *also show no evidence of transcription-associated mutational biases [15].

Second, the marked reduction in GC content of exonic sequences in *D. saltans *and *willistoni *could also result from a relaxation of natural selection on codon bias in these lineages. This could in principle result from a reduction in effective population size, but this model would require a severe and prolonged bottleneck in the lineage leading to the *saltans/willistoni *species group, or concomitant reductions in effective population size for all species in this group. Alternatively, weaker selection on codon bias in the *saltans/willistoni *group could reflect lineage-specific fitness effects for synonymous mutations. As is the case in *D. saltans *and *D. willistoni*, several other species in the *D. melanogaster *subgroup show significant changes in codon bias in the absence of marked base-composition heterogeneity of nearby noncoding sequences, which has been argued to result from either a relaxation of selection on codon bias mediated through lineage-specific changes in effective population size, from fitness effects, [10,40] or from positive selection [41].

Like the changing codon preference model, a general model of relaxation of selection on codon bias in *D. saltans/willistoni *would also predict that the genes with the highest codon bias would experience the greatest deviation in codon usage in *D. saltans/willistoni *relative to *D. melanogaster*. Formally distinguishing between the shifting codon preference and relaxation of selection models by specifically comparing codon usage preferences in lowly and highly expressed genes between *D. melanogaster *and *D, willistoni *at a larger scale will soon become possible, once the *D. willistoni *genome has been assembled and annotated.

## Conclusion

Analysis of substitutional patterns inferred from nonfunctional fragments of transposable elements in the *Drosophila saltans/willistoni *lineage provides evidence in support of a minor shift in patterns of nucleotide substitution towards decreased GC content of at most 11.5%. These novel patterns of nucleotide substitution are likely modulating the base composition of unconstrained sequences in the genome, and are sufficient to account for documented shift in GC content of noncoding sequences such as introns. The base composition of sequences under selective constraint such as coding sequences, however, while governed in part by substitutional biases, appear to be primarily modulated by other evolutionary forces. In this particular instance, one likely explanation for the reduction in GC content in coding sequences in the *saltans/willistoni *group is a shift in codon preferences combined with an altered substitutional profile. However, general relaxation of selective pressure on codon bias in this species group is also possible, and further investigation will likely reveal the relative strengths of substitutional biases and natural selection in the determination of base composition of functional sequences.

## Methods

### *Drosophila *strains

Strains of *Drosophila saltans*, *D. tropicalis*, *D. equinoxialis*, *D. paulistorum *and *D. willistoni *were kindly provided by E. Baldal. *D. saltans *and *D. willistoni *samples were collected on the Donato Trail on Barro Colorado Island (BCI), Panama, *D. tropicalis *was collected in old forest in BCI, *D. equinoxialis *was collected on the Foster trail in Gigante, Panama, and *D. paulistorum *was collected from the Summit Botanical Gardens in Panama. We also purchased strains 14041-0831.0, 14042-0841.3, 14044-0872.0, and 14045-0901.0 from the University of Arizona Center for Insect Science Stock center, which correspond to *D. neocordata*, *D. emarginata, D. subsaltans*, and *D. prosaltans*, respectively.

### Genomic DNA extraction

We extracted genomic DNA from single individuals taken from these nine strains of *Drosophila *according to protocol described by Greg Gloor and William Engels (personal communication). Each fly was crushed with the end of a pipette tip and subsequently immersed in a buffered solution (10 mM Tris-HCl pH 8.2, 1 mM EDTA, 25 mM NaCl, 200 μg/mL proteinase K). This was incubated at 37°C for 30 minutes, and then at 95°C for 2 minutes to inactivate the proteinase K.

### Species confirmation

To confirm the species assignment for each of the nine strains, we amplified and sequenced a portion of the *xanthine dehydrogenase *(*Xdh*) gene (*rosy *in *D. melanogaster*). The region of *Xdh *studied is approximately 725 bp in size and includes part of exon II in addition to intron B, the distribution of which is confined to the saltans and *willistoni *lineages [42]. Primer sequences were: XDHF: 5' -CGTTCYTTGGTWGTWAGYC-3' and XDHR: 5' -GCAAAGGCYTCCTCCACATT-3'.

Amplifying conditions for the *Xdh *regions for each of the nine species are as follows: 94°C for 2 min, 35 cycles of 94°C for 30s, 52°C for 30s, 72°C for 30s, and a final extension of 72°C for 7 min. All PCR reactions were 20 μl, and each contained 10 μl ReadyMix REDTaq PCR Reaction Mix with MgCl_2 _(Sigma-Adrich, St Louis, MO, USA), 1 μl of each 20 μM primer, 7 μl H_2_O, and 1 μl genomic DNA. PCR reactions were enzymatically cleaned with exonuclease I and shrimp alkaline phosphatase, and were cycle-sequenced in half-strength half-reactions with Big Dye terminator mix (Applied Biosystems) under standard cycling conditions. These reactions were precipitated using ethanol and sodium acetate and sequenced on an ABI 377 sequencer (Applied Biosystems). Sequences were compared with the available sequences for this region in each species [28], with intron B serving as a diagnostic marker.

### Identification of non-LTR elements

Degenerate primers designed for the reverse transcriptase domain of non-LTR retrotransposable elements in *Arabidopsis *were used to amplify potential retrotransposons in *Drosophila saltans*. Primer sequences were: DVO144: 5' -GGGATCCNGGNCCNGAYGGNWT-3' and DVO145: 5' -GGAATTCGGNSWNARNGGRYMNCCYTG-3' [43]. All PCR reactions were 20 μl, and each contained 10 μl ReadyMix REDTaq PCR reaction mix with MgCl_2 _(Sigma-Aldrich), 0.8 μl of 25 mM MgCl_2_, 1.25 μl of each 20 μM primer, 5.7 μl H_2_O, and 1 μl genomic DNA. Cycling conditions were: 94°C for 2 min, 35 cycles of 94°C for 1 min, 47°C for 1 min, 72°C for 2 min, and a final extension of 72°C for 15 min.

The pool of PCR products was cloned using a TOPO cloning kit (Invitrogen) for sequencing, and PCR (using vector primers T7 and M13R) was used to screen for colonies containing appropriately sized inserts. PCR products of interest were enzymatically cleaned with exonuclease I and shrimp alkaline phosphatase, and were cycle-sequenced in half-strength half-reactions with Big Dye (Applied Biosystems) under standard cycling conditions. These reactions were precipitated using ethanol and sodium acetate and sequenced on an ABI 377 sequencer (Applied Biosystems).

These sequences were compared against sequences of all known non-LTR elements using BLASTX to identify clones containing inserts truly corresponding to retrotransposable elements. This technique yielded one clone containing a 271-bp insert with high homology to reverse transcriptases from other organisms, and has highest homology to the *Q *retrotransposon isolated from *Anopheles gambiae *[29]; specific primers for this putative *Q *element were designed from the sequence of this clone.

### Amplification of non-LTR elements in *saltans *and *willistoni *clades

These element-specific primers were used to amplify paralogous copies of this *Q *element in several species in the *saltans *and *willistoni *clades. Primer sequences are as follows: SaltansQF: 5' -CCGGATGGGATAGCTG-3' and SaltansQR: 5' -GGTTAGCGGTAGTAGATGTA-3'. Amplifications were carried out in 20 μl reactions. For species in the *saltans *clade, each PCR reaction contained 10 μl ReadyMix REDTaq PCR reaction mix with MgCl_2 _(Sigma), 1 μl of each 20-μM element-specific primer, 7 μl H_2_O, and 1 μl genomic DNA. Cycling conditions were: 94°C for 2 min, 35 cycles of 94°C for 1 min, 47°C for 1 min, 72°C for 2 min, and a final extension of 72°C for 15 min.

For species in the *willistoni *clade, two rounds of PCR were required for successful amplification. First, a 20-μl reaction using DVO144 and DVO145 primers was carried out as described above. This PCR product was diluted 1:1000 and was used as template in a second round of PCR amplification using element-specific primers as described for the species in the *saltans *clade.

Products from PCR reactions using element-specific primers were cloned for each of the nine species under study. Colonies of interest were sequenced using T7 and M13R; paralogs differing by <1% within an individual species were not included in the analysis.

### Analysis of background substitutional patterns

In total, 58 distinct paralogous fragments (271 bp in length) of a non-LTR element were identified from the *saltans *and *willistoni *species groups. Sequences have been deposited to Genbank (under accession numbers AY920638-AY920695). We aligned the sequences using Sequencher software (version 3.1.1), and used PAUP software (Phylogenetic Analysis Using Parsimony; version 4.0b9) for the phylogenetic reconstruction of the relationships among the paralogous retrotransposons. Based on this phylogeny, we inferred the ancestral sequence at each terminal node; substitution frequencies were estimated by comparing those ancestral sequences with the sequences of the extant fragments.

The extant sequences show on average 5.2% divergence from the ancestral sequences at their respective terminal nodes on the phylogeny. For the estimation of the substitution frequencies, we used a maximum-likelihood approach to include multiple and back substitutions within one branch at the same site. Importantly, we cannot capture multiple substitutions on different branches. However, owing to the low divergence among paralogs of *Q-saltans1*, the associated error due to such processes is likely to be quite small. All 12 possible single-nucleotide substitutions have been estimated. Details on a more general version of this approach including neighbor-dependent substitution processes have been discussed by Arndt *et al*. [35,36]. Once the 12 substitution frequencies are established, the stationary GC content (GC*) can easily be computed [44].

Because we only have a finite amount of sequence data from which to estimate the substitution frequencies, these estimates will be affected by statistical errors; we can estimate these errors by bootstrapping our dataset. For a category with a total of *n *aligned base pairs we resampled the data, drawing randomly and with replacement, *n *pairs of aligned ancestral and daughter nucleotides. From this resampled sequence data, we estimated the substitution frequencies and the GC content as above. We repeated this resampling procedure *M *times, and from the *M *estimates of the above quantities calculated their standard deviation, which gave the statistical error due to the limited amount of sequence data. In our case, we found that *M *= 500 samples is sufficient to estimate those errors [36].

### Background substitutional patterns in *D. melanogaster*

Substitution patterns from *D. melanogaster *were inferred in three ways. Two profiles are based on autosomal copies of the element *DNAREP1_DM *[15], and we present data from elements found in both heterochromatic and euchromatic sequences. Although initial analysis of background substitutional patterns using *DNAREP1_DM *did distinguish between elements in high- versus low-recombination areas of the genome, there were no significant differences revealed between these two genomic locations [15]. As a result, these data were combined in the present analyses and are referred to as "euchromatic" elements. The pattern of substitutions does significantly differ between heterochromatic and euchromatic regions in *D. melanogaster*, but the differences are very small in magnitude [15].

We also present data from the retrotransposon *Helena *[45], which is based on 22 paralogs of this transposable element. These data were analyzed in the same way as the data from *D. saltans *and *willistoni*; phylogenies were reconstructed using PAUP, ancestral sequences at each terminal node were inferred, and substitution frequencies were estimated by comparing those ancestral sequences with the sequences of the extant fragments in a maximum-likelihood framework. Details on a more general version of the maximum-likelihood model used to estimate substitutional patterns in *D. melanogaster *including neighbor-dependent substitution processes have been discussed by Arndt *et al*. [35].

## Authors' contributions

NDS, PFA, and DAP contributed to the development of this project idea, and the analysis and interpretation of the data presented in this report. NDS, PFA, and DAP were also responsible for the writing and editing of this manuscript, and have all given their final approval for its publication.

## Figures and Tables

**Table 1:** Relative rates of single-nucleotide substitutions (with standard errors) in *D. melanogaster *and *D. saltans/willistoni*

	T:A->A:T	G:C->C:G	T:A->G:C	G:C->T:A	T:A->C:G	C:G->T:A
*D. melanogaster (*Heterochromatic* DNAREP1_DM)*	0.15 (0.004)	0.10 (0.003)	0.11 (0.003)	0.21 (0.005)	0.13 (0.003)	0.30 (0.005)
*D. melanogaster (*Euchromatic *DNAREP1_DM)*	0.14 (0.002)	0.09 (0.002)	0.11 (0.002)	0.21 (0.003)	0.15 (0.002)	0.30 (0.004)
*D. saltans/willistoni (Q-saltans1)*	0.10 (0.016)	0.09 (0.020)	0.05 (0.012)	0.15 (0.026)	0.18 (0.021)	0.43 (0.043)

**Table 2:** Ratio of rates of significantly different single-nucleotide substitutions between *D. saltans/willistoni *and *D. melanogaster*

	T:A->C:G r	C:G->T:A	Minimum fixation bias*	T:A->G:C	G:C->T:A	Minimum fixation bias*
Ratio of rates in *saltans/willistoni *and *D. melanogaster (*Euchromatic * DNAREP1_DM)*	1.23 (0.13)**	1.43 (0.12)	*N*_*e*_*s*(mel) = -4.76 *N*_*e*_*s*(saltans) = -4.85	0.47 (0.10)	0.71 (0.11)	*N*_*e*_*s*(mel) = 2.86 *N*_*e*_*s*(saltans) = 2.98
Ratio of rates in *saltans/willistoni *and *D. melanogaster (*Heterochromatic *DNAREP1_DM)*	1.33 (0.13)	1.43 (0.12)	undefined	0.21 (0.003)	0.15 (0.002)	undefined

* Fixation bias is measured in the effective strength of natural selection (product of effective population size and selection coefficient) that would generate such a bias. Positive sign implies preference for A:T pairs and negative sign implies preference toward G:C pairs.

** The number in parentheses is the standard error.

**Table 3:** Percent GC of coding and noncoding sequences from *D. willistoni*, *D. saltans *and *D. melanogaster*

	*Adh*^a^	*Sod*^a^	*Per*	*Xdh*^a^	*Amyrel*^a^	*Ddc*^a^	*Gpdh*^a^	Short Introns	Long Introns
*D. saltans*	52.6	45.9	54.9^b^	43.5				27.1^c^	30.6^c^
*D. willistoni*	54.1	52.1	60.5^a^	47.2	48.5	52.9	51.6	32.9^c^	35.5^c^
*D. melanogaster*	81.5	76.0	80.8^a^	64.4	68.2	73.6	76.5	34.5^d^	37.1^d^

^a^Percent GC at third position sites as reported by Rodriguez-Trelles *et al*. [22]

^b^Percent GC at third position sites as reported by Rodriguez-Trelles *et al*. [23]

^c^Percent GC from combined analysis of *RpL32*, *Adh*, *Hsp83*, *Sod*, *Gpdh*, *Amyrel*, *Ddc*, *per *and *ry*, as reported by Powell *et al*. [27]

^d^Percent GC from 225 introns in *D. melanogaster*, as reported by Hadrill *et al*. [38]
